# Prevalence and risk factors of myocardial and acute kidney injury following radical nephrectomy with vena cava thrombectomy: a retrospective cohort study

**DOI:** 10.1186/s12871-021-01462-y

**Published:** 2021-10-12

**Authors:** Yi-Bin Hua, Xue Li, Dong-Xin Wang

**Affiliations:** 1 Department of Anesthesiology and Critical Care Medicine, Peking University First Hospital, Beijing, 100034 China; 2 Outcomes Research Consortium, Cleveland, OH USA

**Keywords:** Renal cell carcinoma, Radical nephrectomy, Inferior vena cava thrombectomy, Myocardial injury, Acute kidney injury

## Abstract

**Background:**

Radical nephrectomy with thrombectomy is the mainstay treatment for patients with renal cell carcinoma with vena cava thrombus. But the procedure is full of challenge, with high incidence of major complications and mortality. Herein, we investigated the incidence and predictors of myocardial injury and acute kidney injury (AKI) in patients following radical nephrectomy with inferior vena cava thrombectomy.

**Methods:**

Patients who underwent nephrectomy with thrombectomy between January 2012 and June 2020 were retrospectively reviewed. Myocardial injury was diagnosed when peak cardiac troponin I was higher than 0.03 ng/ml. AKI was diagnosed according to the Kidney Disease: Improving Global Outcomes (KDIGO) criteria. Multivariable logistic regression models were used to identify predictors of myocardial injury or AKI after surgery.

**Results:**

A total of 143 patients were included in the final analysis. Myocardial injury and AKI occurred in 37.8 and 42.7% of patients after this surgery, respectively. Male sex (odds ratio [OR] 0.27, 95% confidence interval [CI] 0.10–0.71; *P* = 0.008) was associated with a lower risk, whereas high level Mayo classification (compared with Mayo level I + II, Mayo level III + IV: OR 4.21, 95% CI 1.42–12.4; *P* = 0.009), acute normovolemic hemodilution before surgery (OR 2.66, 95% CI 1.10–6.41; *P* = 0.029), long duration of intraoperative tachycardia (per 20 min: OR 1.49, 95% CI 1.10–2.16; *P* = 0.036), and long duration of surgery (per 1 h, OR 1.48, 95% CI 1.03–2.16, *P* = 0.009) were associated with a higher risk of myocardial injury. High body mass index (OR 1.18, 95% CI 1.06–1.33; *P* = 0.004) and long duration of intraoperative hypotension (per 20 min: OR 1.30, 95% CI 1.04–1.64; *P* = 0.024) were associated with a higher risk, whereas selective renal artery embolism before surgery (OR 0.20, 95% CI 0.07–0.59, *P* = 0.004) was associated with a lower risk of AKI.

**Conclusion:**

Myocardial injury and AKI were common in patients recovering from radical nephrectomy with inferior vena cava thrombectomy. Whether interventions targeting the above modifiable factors can improve outcomes require further studies.

**Supplementary Information:**

The online version contains supplementary material available at 10.1186/s12871-021-01462-y.

## Background

Renal cell carcinoma (RCC) has a propensity to develop local extension into the venous system. About 4 to 10% of newly diagnosed RCC cases have venous tumor thrombus [1–4]. In a study of 540 patients with RCC and venous tumor thrombus, 64.6% had renal venous thrombus (Mayo level 0) and 35.4% had inferior vena cava (IVC) thrombus (12.2% Mayo level I, 14.3% Mayo level II, 5.2% Mayo level III, and 3.7% Mayo level IV, respectively) [5]. For patients with RCC and IVC tumor thrombus, radical nephrectomy with thrombectomy remains the mainstay treatment and offers reasonable long-term survival [6–8]. However, perioperative management of such patients is a great challenge for both surgeons and anesthesiologists [9–12]. Previous studies reported that major complications occurred in 6 to 34% and mortality occurred in 0 to 10.5% of patients following radical nephrectomy with IVC thrombectomy [13–15].

As a challenging surgery, nephrectomy with IVC thrombectomy may also put patients at risk of myocardial and acute kidney injury (AKI). Myocardial injury is defined as troponin elevation [16] and occurred in 8 to 16% patients after non-cardiac surgery [17, 18]. Although usually asymptomatic and without electrocardiographic and imagining changes [19], the occurrence of myocardial injury is associated with worse outcomes including increased 30-day and 1-year mortality [18, 20, 21]. AKI, characterized by oliguria and increased serum creatinine and other biomarkers [22, 23], is also common after major non-cardiac surgery with an incidence from 6.8 to 39.3% [24, 25] and up to 61.6% after radical nephrectomy [26]. Development of postoperative AKI is associated with prolonged hospital stay, long-term decline of renal function, and high mortality [27–29]. However, few studies have focused on the incidence and risk factors of myocardial injury and AKI after radical nephrectomy with IVC thrombectomy. In a small sample size study of 76 patients, 53.9% developed AKI after surgery; long IVC clamping time was identified as a potentially modifiable risk factor [30].

A better understanding of the occurrence and underlying risk factors of myocardial injury and AKI may help us to prevent these harmful complications after surgery for RCC and IVC thrombus. This retrospective study aimed to investigate the incidence and risk factors of myocardial injury and AKI in patients undergoing radical nephrectomy with IVC thrombectomy.

## Methods and materials

### Ethics and consent

This retrospective cohort study was performed in Peking University First Hospital. The study protocol was approved by the Biomedical Research Ethics Committee of Peking University First Hospital (2019–205). Considering that all data were collected from the hospital electronic medical record system and no patient follow-ups were performed, the Ethics Committee agreed to waive written informed consents. All personal data were kept strictly confidential. The study was performed in accordance with Strengthening the Reporting of Observational Studies in Epidemiology (STROBE) criteria (see Additional file 1: STROBE checklist).

### Study population

Patients who underwent radical nephrectomy with tumor thrombectomy from January 2012 to June 2020 were screened for study inclusion. The inclusion criteria were adult (age ≥ 18 years) patients who were diagnosed with RCC and IVC tumor thrombus (i.e., Mayo levels I to IV) and underwent radical nephrectomy with IVC thrombectomy. Patients were excluded if they had incomplete data for primary outcome assessment (i.e., no serum troponin or creatinine test results after surgery) in the electronic medical records, were classified as Mayo level 0 (tumor thrombus limited to the renal vein), underwent concomitant cardiac surgery, or turned out to be non-renal carcinomas according to postsurgical pathological report.

### Perioperative management

All patients received contrast-enhanced abdominal computed tomography or magnetic resonance imaging within about 2 weeks before surgery. The thrombus level was classified according to the Mayo classification: Level 0 indicates tumor thrombus limited within the renal vein; Level I, tumor thrombus extending into the IVC to no more than 2 cm above the renal vein; level II, thrombus extending into the IVC to more than 2 cm above renal vein but below the hepatic veins; level III, thrombus extending into the IVC to above the hepatic vein but not to the diaphragm; and level IV, thrombus extending into the supradiaphragmatic IVC or right atrium [5]. Selective renal arterial embolization was performed preoperatively depending on patients’ condition.

In the operating room, routine monitoring included electrocardiogram (ECG), non-invasive blood pressure, pulse oxygen saturation, end-tidal concentration of carbon dioxide and inhaled anesthetics, nasopharyngeal temperature, Bispectral Index, and urine output. Invasive blood pressure was monitored through an intra-arterial cannula with or without dynamic parameter (such as stroke volume variation or pulse pressure variation) monitoring. A central venous line was established through which acute normovolemic hemodilution was performed after anesthesia induction when considered necessary. For patients with tumor thrombi of Mayo level III or above, transesophageal echocardiogram was used to monitor the position of the tumor thrombus.

General anesthesia was performed for all patients. Anesthesia was induced with intravenous propofol/etomidate, opioids (sufentanil/remifentanil) and muscle relaxants (rocuronium or cisatracurium), and maintained with intravenous infusion of propofol and sufentanil/remifentanil, with or without inhalational nitrous oxide and/or sevoflurane. Muscle relaxation was maintained with rocuronium or cisatracurium. Regional block was performed and dexmedetomidine was administered at the discretion of anesthesiologists. Acute normovolemic hemodilution was performed after anesthesia induction but before surgical incision. The volume of collected blood was calculated so that the hematocrit was maintained above 27% after hemodilution [31]. Balanced crystalloid fluid was routinely infused. Normal saline was only used as a carrier for antibiotics and other drugs. For patients with large blood loss, artificial colloid was infused for volume resuscitation; blood products were transfused in order to maintain hemoglobin > 7 g/dl and normal coagulation. The target of hemodynamic management was to maintain blood pressure and heart rate within 30% from baseline and a urine output > 0.5 ml/kg/h.

Surgery was performed via laparoscopic, open or combined approaches, depending on the condition of tumor and the decision of surgeons. For patients with Mayo level I thrombi, surgeries were usually performed under partial IVC clamping. Patients with Mayo level II thrombi usually required complete clamping of IVC and renal vein. For patients with tumor thrombi of Mayo level III or above, additional cross-clamping of hepatic hilar might be necessary or cardiopulmonary bypass (CPB) was performed with systemic heparinization. Due to the nature of cancer surgery, intraoperative blood salvage was not performed unless for patients with massive bleeding, during which case the salvaged blood would be transfused after obtaining written informed consents.

After surgery, patients with intraoperative hemodynamic instability, massive bleeding, or CPB were admitted to the intensive care unit (ICU); otherwise, they were admitted to the post-anesthesia care unit for at least 30 min and then transferred back to the general wards. Crystalloid solutions (containing electrolytes, glucose, and other non-electrolyte solutes) were infused. Blood products (packed red blood cells, fresh frozen plasma, and/or albumin) were administered as necessary. As a routine practice, serum levels of cardiac troponin I (cTnI) and creatinine were monitored at least once during the first three postoperative days or longer when considered necessary. Patient-controlled analgesia (PCA) was provided for postoperative analgesia. Nonsteroidal anti-inflammatory drugs were allowed for those without contraindications. Other postoperative care was provided per routine.

### Data acquisition and outcomes

Data were collected from the electronic medical record system of the hospital. Baseline data included demographic characteristics, comorbidities, smoking and surgical history, main laboratory test results (including baseline cTnI and creatinine), and location and maximal diameter of the tumor. Charlson Comorbidity Index was calculated. American Society of Anesthesiologists classification and New York Heart Association classification were evaluated. Mayo classification was obtained from surgical records.

Intraoperative data included the conduct of selective renal arterial embolization, type and duration of anesthesia, medication during anesthesia, performance of ANH, intraoperative levels of hemoglobin and lactic acid, occurrence of hemodynamic fluctuation, fluid infusion, blood loss and allogeneic blood transfusion, urine output, type and duration of surgery, combined surgery, use of IVC clamping and hepatic hilar clamping, use of CPB, as well as administration of PCA after surgery. Hemodynamic data were obtained from the anesthesia information system, which captured parameters automatically every 10 s throughout the intraoperative period.

The primary outcomes were myocardial injury and AKI after surgery. The results of serum cTnI and creatinine during early postoperative days were collected. Myocardial injury was diagnosed when peak cTnI was higher than the 99th percentile upper reference limit (> 0.03 ng/ml) or, for patients with preoperative cTnI above normal, an absolute increase of ≥0.03 ng/ml [16–18]. Acute myocardial infarction (AMI) was diagnosed when myocardial injury was accompanied by clinical evidence of acute myocardial ischemia (symptoms, ECG changes, or imaging findings) [16]. AKI was diagnosed according to the Kidney Disease: Improving Global Outcomes (KDIGO) criteria [32], i.e., an increase in serum creatinine ≥26.5 μmol/l (≥0.3 mg/dl) within 48 h, or an increase in serum creatinine to ≥1.5 times baseline within 7 days after surgery. For patients who developed AKI, the severity was classified into 3 stages. Stage 1 indicates 1.5 to 1.9 times baseline or ≥ 26.5 μmol/l increase; stage 2 indicates 2.0 to 2.9 times baseline; stage 3 indicates 3.0 times baseline or an increase in serum creatinine to ≥353.6 μmol/l or requirement of renal replacement therapy [32].

Other postoperative outcomes, including ICU admission after surgery, the development of other postoperative complications, length of hospital stay after surgery, and in-hospital mortality were also collected. For patients admitted to the ICU, the duration of mechanical ventilation and length of ICU stay were also recorded.

### Statistical analyses

Continuous variables are presented as the mean ± standard deviation or median (interquartile range [IQR]). Data were compared using the student’s t test (normal distribution) or Mann–Whitney U test (non-normal distribution). Categorical variables are presented as number of patients (percentage). Data were analyzed using the Chi-squared test, Chi-square test with continuity correction or Fisher’s exact test. Time-to-event variables are presented as median (95% CI). Data were analyzed using Kaplan-Meier survival analysis, with difference between group compared with log-rank test. Missing data were not replaced.

Univariate logistic regression analyses were performed to screen factors in association with the occurrence of myocardial injury and AKI. Linear regression was used to test the multicollinearity among variables, a variance inflation factor of > 10 was considered the existence of multicollinearity. Independent variables with *P* < 0.20 in univariable analyses or were considered clinically important were included in multivariable logistic regression models to identify factors that were significantly associated with the occurrence of myocardial injury or AKI after surgery. Hosmer-Lemeshow test was used to assess the goodness-of-fit of the models. A two-sided *P* value of < 0.05 was considered statistically significant. All data were analyzed using SPSS (version 25.0; IBM SPSS, Inc., Chicago, IL, USA).

We did not estimate sample size in advance. However, according to the “ten events per variable” rule and the number of independent variables included in the final multivariable logistic regression models, the number of patients with outcome events was sufficient to guarantee the stability of the regression estimates.

## Results

### Patients

**Fig. 1:**
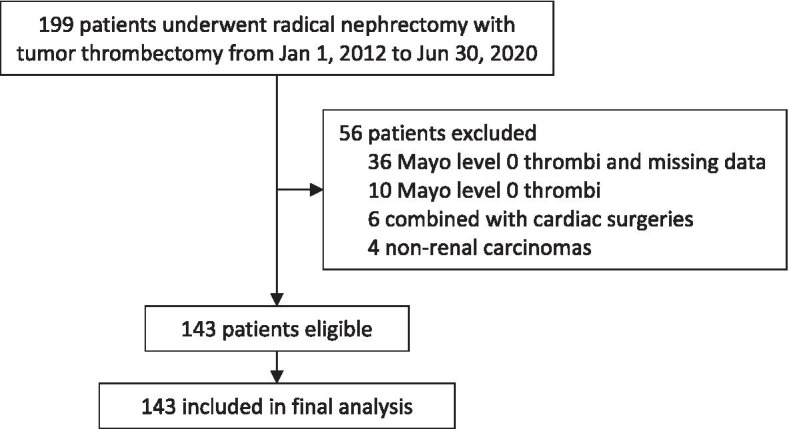
Flowchart of the study

A total of 199 patients underwent radical nephrectomy with tumor thrombectomy from January 1, 2012 to June 30, 2020. Among these, 36 patients were excluded due to Mayo level 0 thrombi and missing data, 10 patients were excluded due to the Mayo level 0 thrombi, 6 patients were excluded for concomitant cardiac surgeries, and 4 patients were excluded for non-renal carcinomas. The remaining 143 patients were included in the final analysis (Fig. 1).

When compared with patients without myocardial injury, those who developed myocardial injury had lower male sex ratio, higher baseline cTnI level, and higher percentage with Mayo level IV thrombi (Table 1). During anesthesia, those who developed myocardial injury received less nitrous oxide inhalation but more ANH, had lower hemoglobin but higher lactic acid level, developed more and longer hypotension and tachycardia; they underwent longer anesthesia and surgery, received more fluid infusion and blood products, had more urine output and blood loss, endured more IVC/hepatic hilum clamping and CPB, and were given more PCA (Table 2).

**Table 1:** Baseline data

	Non-myocardial injury (*n* = 89)	Myocardial injury (*n* = 54)	*P* value	Non-AKI (*n* = 82)	AKI (*n* = 61)	*P* value
Age, year	59 (50, 67)	55 (48, 64)	0.092	56 (44, 68)	58 (46, 70)	0.433
Body mass index, kg/m^2^	24.2 (22.6, 27.1)	23.9 (21.6, 26.8)	0.441	24.0 (20.7, 27.3)	25.9 (19.6, 32.2)	**0.021**
Male sex	71 (79.8%)	34 (63.0%)	**0.027**	63 (76.8%)	42 (68.9%)	0.286
Comorbidities						
Stroke	3 (3.4%)	4 (7.4%)	0.278	2 (2.4%)	5 (8.2%)	0.115
Hypertension	36 (40.4%)	22 (40.7%)	0.973	31 (37.8%)	27 (44.3%)	0.437
Chronic heart disease ^a^	6 (6.7%)	6 (11.1%)	0.361	6 (7.3%)	6 (9.8%)	0.591
Diabetes Mellitus	16 (18.0%)	9 (16.7%)	0.841	17 (20.7%)	8 (13.1%)	0.236
COPD	4 (4.5%)	0 (0.0%)	0.290	4 (4.9%)	0 (0.0%)	0.216
Elevated transaminase ^b^	6 (6.7%)	5 (9.3%)	0.584	7 (8.5%)	4 (6.6%)	0.660
Abnormal kidney function ^c^	9 (10.1%)	2 (3.7%)	0.163	8 (9.8%)	3 (4.9%)	0.283
Smoking history	29 (32.6%)	18 (33.3%)	0.926	25 (30.5%)	22 (36.1)	0.482
Previous surgery	15 (16.9%)	10 (18.5%)	0.799	18 (22.0%)	7 (11.5%)	0.103
Charlson Comorbidity Index ^d^	2 (2, 2)	2 (2, 2)	0.748	2 (2, 2)	2 (2, 2)	0.931
Laboratory tests						
Hemoglobin, g/L	119 (105, 135)	110 (98, 130)	0.429	112 (96, 134)	117 (108, 131)	0.327
Albumin, g/L	37.8 (35.1, 41.2)	36.5 (33.8, 40.7)	0.227	37.6 (35.1, 41.4)	37.6 (34.4, 40.8)	0.728
cTnI, ng/ml	0.004 (0.001, 0.010) [63]	0.010 (0.007, 0.010) [27]	**0.026**	0.010 (0.003, 0.010) [56]	0.007 (0.001, 0.010) [34]	0.296
cTnI > 0.03 ng/ml ^e^	2 (2.2%)	2 (3.7%)	> 0.999	2 (2.4%)	2 (3.3%)	0.969
Creatinine, μmol/L	96.0 (80.3, 114.0)	90.5 (80.1, 105.3)	0.422	101.0 (83.0, 117.0)	88.3 (74.2, 104.0)	**0.004**
ASA classification			0.095			0.732
I	8 (9.0%)	1 (1.9%)		5 (6.1%)	4 (6.6%)	
II	59 (66.3%)	32 (59.3%)		54 (65.9%)	37 (60.7%)	
III	22 (24.7%)	20 (37.0%)		22 (26.8%)	20 (32.8%)	
IV	0 (0%)	1 (1.9%)		1 (1.2%)	0 (0%)	
NYHA classification			0.407			0.531
I	77 (86.5%)	47 (87.0%)		70 (85.4%)	54 (88.5%)	
II	12 (13.5%)	6 (11.1%)		12 (14.6%)	6 (9.8%)	
III	0 (0%)	1 (1.9%)		0 (0%)	1 (1.6%)	
Right renal tumor (vs. left)	61 (68.5%)	42 (77.8%)	0.233	60 (73.2%)	43 (70.5%)	0.724
Maximal tumor diameter, cm			0.261			0.088
0 to 5	8 (9.0%)	3 (5.6%)		6 (7.3%)	5 (8.2%)	
> 5 to 10	42 (47.2%)	33 (61.1%)		37 (45.1%)	38 (62.3%)	
> 10	39 (43.8%)	18 (33.3%)		39 (47.6%)	18 (29.5%)	
Mayo classification ^f^			**< 0.001**			0.531
I	18 (20.2%)	4 (7.4%)		14 (17.1%)	8 (13.1%)	
II	27 (30.3%)	2 (3.7%)		19 (23.2%)	10 (16.4%)	
III	40 (44.9%)	23 (42.6%)		35 (42.7%)	28 (45.9%)	
IV	4 (4.5%)	25 (46.3%)		14 (17.1%)	15 (24.6%)	
Interval from contrast-enhanced examination, day ^g^	13 (7, 20)	13 (5, 20)	0.997	12 (4, 18)	14 (7, 24)	0.181

Data are median (interquartile range) or number (%). Numbers in square brackets indicate patients with missing data. *P* values in bold indicate < 0.05

*COPD* chronic obstructive pulmonary injury, *cTnI* cardiac troponin I, *ASA* American Society of Anesthesiologists, *NYHA* New York Heart Association

^a^ Including coronary artery disease, cardiac valve disease, or any type of arrhythmia requiring therapy

^b^ Serum aspartate aminotransferase and/or alanine aminotransferase were higher than the upper normal limit

^c^ Indicating serum creatinine ≥133 μmol/L

^d^ According to the 1987 version without age correction

^e^ Patients without preoperative cardiac troponin I results were regarded as within normal range

^f^ Level I, tumor thrombus extending into the IVC to no more than 2 cm above the renal vein; level II, thrombus extending into the IVC to more than 2 cm above renal vein but below the hepatic veins; level III, thrombus extending into the IVC to above the hepatic vein but not to the diaphragm; and level IV, thrombus extending into the supradiaphragmatic IVC or right atrium

^g^ Time interval from contrast-enhanced examination to surgery

**Table 2:** Intra- and postoperative data

	Non-myocardial injury (*n* = 89)	Myocardial injury (*n* = 54)	*P* value	Non-AKI (*n* = 82)	AKI (*n* = 61)	*P* value
Selective renal arterial embolization	19 (21.3%)	8 (14.8%)	0.333	20 (24.4%)	7 (11.5%)	0.051
Type of anesthesia			0.784			0.229
General	76 (85.4%)	47 (87.0%)		73 (89.0%)	50 (82.0%)	
Combined regional-general ^a^	13 (14.6%)	7 (13.0%)		9 (11.0%)	11 (18.0%)	
Medication during anesthesia						
Nitrous oxide	69 (77.5%)	33 (61.1%)	**0.035**	61 (74.4%)	41 (67.2%)	0.348
Sevoflurane	51 (57.3%)	28 (51.9%)	0.525	41 (50.0%)	38 (62.3%)	0.144
Dexmedetomidine	41 (46.1%)	27 (50.0%)	0.648	43 (52.4%)	25 (41.0%)	0.175
ANH before surgery ^b^	25 (28.1%)	25 (46.3%)	**0.027**	25 (30.5%)	25 (41.0%)	0.193
Hemoglobin after ANH, g/L^c^	98 (86, 110)	102 (94, 109)	0.403	105 (88, 111)	98 (91, 109)	0.466
Lactic acid after ANH, mmol/L^c^	1.0 (0.6, 1.4)	0.8 (0.5, 1.2) [5]	0.292	0.9 (0.5, 1.2) [2]	0.9 (0.7, 1.3) [3]	0.466
Extremes of arterial blood gas analyses						
Lowest hemoglobin, g/L	95 (77, 112)	74 (61, 88)	**< 0.001**	87 (62, 112)	90 (64, 116)	0.632
Highest lactic acid, mmol/L	1.2 (0.9, 1.6)	1.7 (1.0, 2.5)	**0.004**	1.5 (0.6, 2.2)	2.0 (0.1, 3.9)	**0.044**
Highest lactic acid > 2 mmol/L	15 (16.9%)	21 (38.9%)	**0.002**	17 (20.7%)	19 (31.1%)	0.156
Hemodynamic change during anesthesia						
Hypotension ^d^	76 (85.4%)	52 (96.3%)	**0.039**	73 (89.0%)	55 (90.2%)	0.826
Duration of hypotension, min	8 (1, 23)	37 (9, 60)	**< 0.001**	12 (1, 35)	18 (3, 54)	0.141
Tachycardia ^e^	45 (50.6%)	44 (81.5%)	**< 0.001**	46 (56.1%)	43 (70.5%)	0.079
Other tachyarrhythmia ^f^	2 (2.2%)	8 (14.8%)	**0.004**	6 (7.3%)	4 (6.6%)	0.860
Duration of tachycardia, min ^e,f^	0.2 (0.0, 3.3)	6.2 (0.3, 22.2)	**< 0.001**	0.9 (0.0, 8.1)	2.2 (0.0, 9.7)	0.303
Bradycardia ^g^	11 (12.4%)	16 (29.6%)	**0.011**	14 (17.1%)	13 (21.3%)	0.522
Total fluid infused, ml	4000 (2800, 5600)	5550 (4050, 7300)	**0.001**	4800 (3300, 6125)	4900 (2800, 6700)	0.637
Balanced crystalloid, ml	2400 (2000, 3500)	2650 (1925, 3700)	0.289	2575 (2000, 3525)	2500 (1700, 3700)	0.219
Normal saline, ml	100 (100, 100)	100 (100, 200)	0.417	100 (100, 125)	100 (100, 100)	0.542
Hydroxyethyl starch, ml	500 (0, 500)	500 (0, 1000)	0.428	500 (0, 625)	500 (0, 1000)	0.528
Succinylated gelatin, ml	500 (0, 500)	500 (0, 500)	0.503	500 (0, 500)	500 (0, 500)	0.729
Urine output, ml	550 (300, 900)	850 (500, 1750)	**< 0.001**	700 (338, 1263)	600 (300, 1100)	0.336
Estimated blood loss, ml	500 (200, 1000)	1000 (500, 2100)	**< 0.001**	600 (300,1525)	800 (200, 1450)	0.453
Allogeneic blood transfusion	38 (42.7%)	43 (79.6%)	**< 0.001**	44 (53.7%)	37 (60.7%)	0.404
Volume of red blood cells, ml	0 (0, 400)	400 (0, 800)	0.306	100 (0, 800)	400 (0, 800)	0.490
Fresh frozen plasma	28 (31.5%)	34 (63.0%)	**< 0.001**	34 (41.5%)	28 (45.9%)	0.596
Platelets concentrate	2 (2.2%)	21 (38.9%)	**< 0.001**	9 (11.0%)	14 (23.0%)	0.054
Intraoperative fluid balance, ml	2730 (2000, 3930)	3350 (2060, 4100)	0.202	3100 (2080, 3960)	2830 (1900, 4160)	0.636
Duration of anesthesia, h	5.7 (4.4, 7.1)	7.9 (6.5, 9.5)	**< 0.001**	6.6 (4.8, 7.9)	6.4 (4.9, 8.6)	0.909
Type of surgery			0.078			0.081
Laparoscopic	16 (18.0%)	3 (5.6%)		7 (8.5%)	12 (19.7%)	
Open	38 (42.7%)	23 (42.6%)		40 (48.8%)	21 (34.4%)	
Laparoscopic combined open	35 (39.3%)	28 (51.9%)		35 (42.7%)	28 (45.9%)	
Combined with non-renal surgery ^h^	2 (2.2%)	1 (1.9%)	0.873	0 (0%)	3 (4.9%)	**0.042**
Complete IVC clamping	69 (77.5%)	50 (92.6%)	**0.019**	68 (82.9%)	51 (83.6%)	0.914
Hepatic hilum clamping	10 (11.2%)	29 (53.7%)	**< 0.001**	24 (29.3%)	15 (24.6%)	0.534
Use of cardiopulmonary bypass	6 (6.7%)	32 (59.3%)	**< 0.001**	20 (24.4%)	18 (29.5%)	0.493
Duration of cardiopulmonary bypass, min ^i^	0 (0, 0)	21 (0, 31)	**< 0.001**	0 (0, 3.5)	0 (0, 20)	0.478
Duration of surgery, h	4.2 (3.1, 5.5)	6.3 (5.0, 7.7)	**< 0.001**	5.0 (3.5, 5.9)	5.0 (3.5, 7.0)	0.746
Use of PCA after surgery	81 (91.0%)	54 (100.0%)	**0.023**	75 (91.5%)	60 (98.4%)	0.138
Postoperative fluid balance, ml						
Postoperative day 1	660 (−260, 2770)	507 (180, 1200)	0.689	670 (220, 2300)	490 (−260, 1760)	0.135
Postoperative day 2	100 (− 440, 1159)	318 (50, 720)	0.319	320 (−350, 1310)	100 (− 340, 720)	0.193
Use of NSAIDs during perioperative period	53 (59.6%)	12 (22.2%)	**< 0.001**	37 (45.1%)	28 (45.9%)	0.926

Data are number (%), or median (interquartile range). Numbers in square brackets indicate patients with missing data. P values in bold indicate < 0.05

*ANH* acute normovolemic hemodilution, *IVC* inferior vena cava, *PCA* patient-controlled analgesia

^a^ Includes epidural anesthesia or rectus sheath/transversus abdominis plane block

^b^ Performed after anesthesia induction through central venous line

^c^ Results of patients after accomplishment of ANH

^d^ Mean arterial pressure < 65 mmHg

^e^ Heart rate > 100 beats per minute

^f^ New onset arrhythmia requiring therapy, including atrial premature, ventricular premature, atrial fibrillation, etc.

^g^ Heart rate < 50 beats per minute

^h^ Combined with splenectomy or cholecystectomy

^i^ Results of all patients

When compared with patients without AKI, those who developed AKI had higher body mass index (BMI) and lower baseline creatinine level (Table 1). During anesthesia, they had higher lactic acid level and underwent more combined non-renal surgery (mainly splenectomy and cholecystectomy) (Table 2).

### Postoperative outcomes

**Fig. 2:**
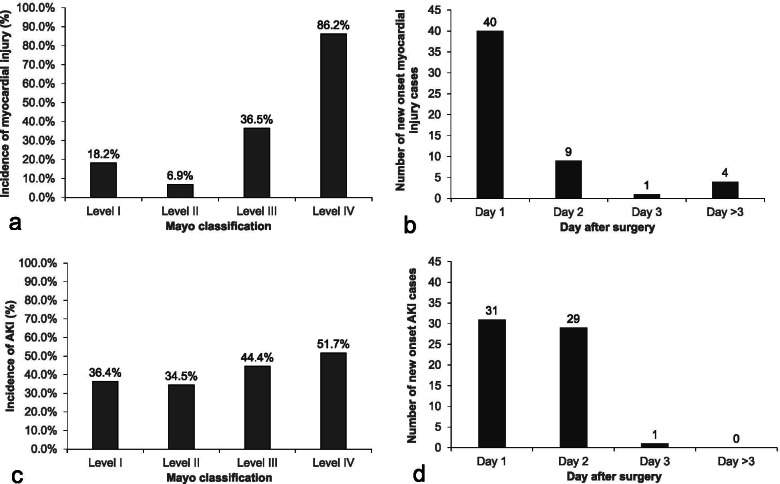
Occurrence of myocardial injury according to Mayo classification level (**a**) and days after surgery (**b**). The majority of myocardial injury occurred in patients with Mayo levels III and IV and during the first day after surgery. Occurrence of acute kidney injury according to Mayo classification level (**c**) and days after surgery (**d**). The development of AKI was not related with Mayo level, but the majority occurred within the first two postoperative days

Of the included patients, 54 (37.8%) developed myocardial injury and 11 (7.7%) developed AMI. The majority of myocardial injury occurred in patients with Mayo levels III and IV thrombi and during the first day after surgery (Fig. 2a and b). When compared with patients without myocardial injury, those with myocardial injury developed more AKI (57.4% [31/54] vs. 33.7% [30/89], *P* = 0.005) and other complications (24.1% [13/54] vs. 1.1% [1/89], *P* = 0.001), had more ICU admission (94.4% [51/54] vs. 64.0% [57/89], *P* < 0.001), and stayed longer in hospital after surgery (hazard ratio [HR] 1.94, 95% CI 1.37–2.74, *P* < 0.001). Among patients admitted to the ICU, those with myocardial injury had more endotracheal intubation (90.7% [49/54] vs. 49.4% [44/89], *P* < 0.001), endured longer mechanical ventilation (HR 2.40, 95% CI 1.67–3.44, *P* < 0.001), and stayed longer in the ICU (HR 2.39, 95% CI 1.68–3.41, *P* < 0.001) than those without (Table 3).

**Table 3:** Postoperative outcomes

	Non-myocardial injury (*n* = 89)	Myocardial injury (*n* = 54)	*P* value	Non-AKI (*n* = 82)	AKI (*n* = 61)	*P* value
Highest cTnI (ng/ml), median (95%CI)	0.010 (0.003, 0.010)	0.790 (0.067, 2.272)	**< 0.001**	0.010 (0.003, 0.038)	0.040 (0.010, 1.100)	**0.004**
Development of myocardial injury	0 (0%)	54 (100.0%)	–	23 (28.0%)	31 (50.8%)	**0.005**
Acute myocardial infarction ^a^	0 (0%)	11 (20.4%)	**< 0.001**	3 (3.7%)	8 (13.1%)	**0.036**
Highest creatinine (μmol/L)	113.0 (98.4, 129.4)	125.0 (99.0, 146.3)	0.152	106.5 (92.0, 123.3)	129.6 (116.1, 160.7)	**< 0.001**
Development of AKI	30 (33.7%)	31 (57.4%)	**0.005**	0 (0.0%)	61 (100.0%)	**–**
Stage of AKI			**0.014**			**–**
Stage 1	26 (29.2%)	27 (50.0%)		0 (0.0%)	53 (86.9%)	
Stage 2	2 (2.2%)	4 (7.4%)		0 (0.0%)	6 (9.8%)	
Stage 3	2 (2.2%)	0 (0.0%)		0 (0.0%)	2 (3.3%)	
Requirement of RRT	1 (1.1%)	0 (0.0%)	> 0.999	0 (0.0%)	1 (1.6%)	0.427
ICU admission	57 (64.0%)	51 (94.4%)	**< 0.001**	59 (72.0%)	49 (80.3%)	0.249
With endotracheal intubation	44 (49.4%)	49 (90.7%)	**< 0.001**	53 (64.6%)	40 (65.6%)	0.907
Mechanical ventilation, h	0 (0, 0)	11 (8, 14)	**< 0.001**	4 (2, 6)	5 (3, 7)	0.247
Length of stay in ICU, h	14 (12, 16)	46 (29, 63)	**< 0.001**	20 (17, 23)	36 (19, 53)	**0.046**
Other complications	1 (1.1%)	13 (24.1%)	**0.001**	2 (2.4%)	12 (19.7%)	**0.005**
New onset arrhythmia ^b^	0 (0.0%)	6 (11.1%)	**0.005**	1 (1.2%)	5 (8.2%)	0.102
Pulmonary embolism ^c^	0 (0.0%)	2 (3.7%)	0.141	0 (0.0%)	2 (3.3%)	0.180
Pulmonary infection ^d^	1 (1.1%)	4 (7.4%)	0.130	1 (1.2%)	4 (6.6%)	0.208
Wound infection ^e^	0 (0%)	1 (1.9%)	0.378	0 (0.0%)	1 (1.6%)	0.427
Length of hospital stay, day	7 (6, 7)	13 (12, 14)	**< 0.001**	8 (7, 9)	10 (7, 13)	**0.027**
In-hospital mortality	0 (0%)	0 (0%)	–	0 (0%)	0 (0%)	–

Data are median (interquartile range), number (%), or median (95% CI). *P* values in bold indicate < 0.05

*AKI* acute kidney injury (diagnosed according to KDIGO criteria), *RRT* renal replacement therapy, *ICU* intension care unit

^a^ Acute myocardial infarction refers to dynamic elevations of cardiac troponin in combination with either ischemic symptoms, electrocardiogram changes, or imaging findings

^b^ New onset arrhythmia refers to new

-onset atrial fibrillation or paroxysmal supraventricular tachycardia necessitating medical treatment

^c^ Pulmonary embolism confirmed by computed tomography pulmonary angiography

^d^ New infiltrate on chest radiograph with hyperthermia (> 38.3 °C) and leukocytosis (≥12,000/mm^3^) and required intravenous antibiotic therapy

^e^ Wound infection refers to pus expressed from the incision and bacteria cultured from the pus

Of the included patients, 61 (42.7%) developed AKI; of these, 53 (86.9%) had stage 1, 6 (9.8%) had stage 2, and 2 (3.3%) had stage 3 AKI. The occurrence of AKI was not related with Mayo level of thrombi, but the majority occurred within the first two postoperative days (Fig. 2c and d). When compared with patients without AKI, patients with AKI developed more myocardial injury (50.8% [31/61] vs. 28.0% [23/82], *P* = 0.005), more AMI (13.1% [8/61] vs. 3.7% [3/82], *P* = 0.036), and more other complications (19.7% [12/61] vs. 2.4% [2/82], *P* = 0.005), and stayed longer in hospital after surgery (HR 1.47, 95% CI 1.04–2.06, *P* = 0.027). Although ICU admission rate did not differ between patients with or without AKI, those with AKI stayed longer in the ICU after surgery (HR 1.41, 95% CI 1.01–1.98, *P* = 0.046; Table 3).

### Predictors of myocardial injury

Univariable analyses identified 25 factors with *P* values < 0.20 (see Additional file 2: Supplement Table). After testing of multicollinearity, 12 factors were included in the multivariable logistic regression model. Final analysis identified five independent predictors of myocardial injury after surgery. Of these, male sex (OR 0.27, 95% CI 0.10–0.71; *P* = 0.008) was associated with a lower risk, whereas high Mayo level (compared with Mayo level I + II, Mayo level III + IV: OR 4.21, 95% CI 1.42–12.4; *P* = 0.009), acute normovolemic hemodilution before surgery (OR 2.66, 95% CI 1.10–6.41; *P* = 0.029), long duration of intraoperative tachycardia (per 20 min: OR 1.49, 95% CI 1.03–2.16; *P* = 0.036), and long duration of surgery (per 1 h, OR 1.48, 95%CI 1.03–2.16; *P* = 0.009) were associated with a higher risk of myocardial injury (Table 4).

**Table 4:** Predictors of myocardial injury

	Univariable analysis ^a^		Multivariable analysis ^b^	
	OR (95% CI)	*P* value	OR (95% CI)	*P* value
Age, year	0.97 (0.95, 1.00)	0.069	–	–
Male sex	0.43 (0.20, 0.92)	0.029	0.27 (0.10, 0.71)	0.008
ASA class (III + IV vs. I + II)			–	–
I + II	Ref		–	–
III + IV	1.94 (0.94, 4.02)	0.075	–	–
Mayo classification				
I + II	Ref		Ref	–
III + IV	8.18 (3.18, 21.1)	< 0.001	4.21 (1.42, 12.4)	0.009
Use of nitrous oxide during anesthesia	0.46 (0.22, 0.95)	0.037	–	–
Acute normovolemic hemodilution before surgery	2.21 (1.09, 4.48)	0.028	2.66 (1.10, 6.41)	0.029
Duration of intraoperative hypotension, 20 min	1.37 (1.11, 1.69)	0.004	–	–
Duration of intraoperative tachycardia, 20 min	1.73 (1.18, 2.53)	< 0.001	1.49 (1.03, 2.16)	0.036
Intraoperative bradycardia	2.99 (1.26, 7.06)	0.013	–	–
Intraoperative allogeneic blood transfusion	5.25 (2.40,11.49)	< 0.001	–	–
Complete IVC clamping during surgery	3.62 (1.17, 11.26)	0.026	–	–
Duration of surgery, h	1.71 (1.37, 2.12)	< 0.001	1.48 (1.03, 2.16)	0.009
Use of NSAIDs during perioperative period	0.19 (0.09, 0.42)	< 0.001	–	–

Preoperative abnormal kidney function and Charlson Comorbidity Index were excluded because of correlation with ASA class

Intraoperative lowest hemoglobin was excluded because of correlation with intraoperative allogeneic blood transfusion

Intraoperative hypotension and intraoperative highest lactic acid were excluded because of correlation with duration of intraoperative hypotension

Intraoperative tachycardia was excluded because of correlation with duration of intraoperative tachycardia

Duration of anesthesia, volume of fluid infusion, intraoperative infusion balance and urine output were excluded because of correlation with duration of surgery

Colloid fluid, fresh frozen plasma and platelet concentrate were excluded because of correlation with the allogeneic blood transfusion

Hepatic hilum clamping and use of cardiopulmonary bypass during surgery and duration of cardiopulmonary bypass were excluded because of correlation with the Mayo classification of tumor thrombus

^a^ Myocardial injury was modeled as a function of a single factor in the univariable logistic regression analysis

^b^ Myocardial injury was modeled as a function of all factors with a *P* value < 0.2 in the univariate analysis or those that were considered clinically important. Multivariable analysis was performed using the backward method. Hosmer-Lemeshow test of goodness of fit of the model: χ^2^ = 3.135, df = 8, *P* = 0.926

### Predictors of AKI

Univariable analyses identified 13 factors with *P* values < 0.20 (see Additional file 2: Supplement Table). After testing of multicollinearity, 12 factors together with age and Mayo classification were included in the multivariable logistic regression model. Final analysis identified three independent predictors of AKI after surgery. Of these, high BMI (OR 1.18, 95% CI 1.06–1.33; *P* = 0.004) and long duration of intraoperative hypotension (per 20 min: OR 1.30, 95% CI 1.04–1.64; *P* = 0.024) were associated with a higher risk, whereas selective renal artery embolism before surgery (OR 0.20, 95% CI 0.07–0.59; *P* = 0.005) was associated with a lower risk of AKI (Table 5).

**Table 5:** Predictors of AKI

	Univariable analysis ^a^		Multivariable analysis ^b^	
	OR (95% CI)	*P* value	OR (95% CI)	*P* value
Age, year ^c^	1.01 (0.98, 1.04)	0.431	–	–
Body mass index, kg/ m^2^	1.12 (1.01, 1.23)	0.029	1.18 (1.06, 1.33)	0.004
Previous stroke	3.57 (0.67, 19.07)	0.136	–	–
Previous surgery	0.46 (0.18, 1.19)	0.108	–	–
Mayo classification ^c^				
I + II	Ref	–	–	–
III + IV	1.61 (0.80, 3.26)	0.186	–	–
Interval from contrast-enhanced examination, day	1.02 (0.99, 1.05)	0.144	–	–
Selective renal arterial embolization	0.40 (0.16, 1.02)	0.056	0.20 (0.07, 0.59)	0.004
Use of sevoflurane during anesthesia	1.65 (0.84, 3.25)	0.145	–	–
Use of dexmedetomidine during anesthesia	0.63 (0.32, 1.23)	0.176	–	–
Acute normovolemic hemodilution before surgery	1.58 (0.79, 3.17)	0.194	–	–
Duration of intraoperative hypotension, 20 min	1.27 (1.04, 1.54)	0.021	1.30 (1.04, 1.64)	0.024
Occurrence of intraoperative tachycardia	1.87 (0.93, 3.77)	0.081	–	–
Transfusion of platelet concentrate	2.42 (0.97, 6.03)	0.059	–	–
Use of PCA after surgery	5.60 (0.67, 46.78)	0.112	–	–

Highest lactic acid during surgery was excluded because of correlation with duration of hypotension

^a^ Acute kidney injury was modeled as a function of a single factor in the univariable logistic regression analysis

^b^ Acute kidney injury was modeled as a function of all factors with a *P* value < 0.2 in the univariate analyses or those that were considered clinically important. Multivariable analysis was performed using the backward method. Hosmer-Lemeshow test of goodness of fit of the model: χ^2^ = 4.612, df = 8, *P* = 0.798

^c^ Included because of clinical importance

## Discussion

Results of this retrospective study showed that myocardial injury occurred in 37.8% and AKI occurred in 42.7% of patients following radical nephrectomy with IVC thrombectomy. Patients with myocardial injury or AKI had worse perioperative outcomes, including more other complications and longer length of hospital stay. Male sex was associated with a lower risk, whereas high Mayo level, acute normovolemic hemodilution, long intraoperative tachycardia, and long surgery were associated with a higher of myocardial injury. High BMI and long intraoperative hypotension were associated with a higher risk, whereas preoperative renal artery embolism was associated with a lower risk of AKI.

As far as we know, this is the first study exploring the incidence and risk factors of myocardial injury after radical nephrectomy with IVC thrombectomy. As expected, the incidence of myocardial injury in our patients was higher when compared with the majority of previous results. For example, the VISION study reported an incidence of 8% [17] and Puelacher et al. [18] reported an incidence of 16% of myocardial injury after noncardiac surgery. However, myocardial injury occurred in up to 40.4% of patients after liver transplantation [33], which is close to our results. The high incidence of myocardial injury in our patients can be attributed to the significant hemodynamic fluctuation during surgery. Indeed, among our patients, 83.2% underwent complete IVC clamping, 27.3% underwent hepatic hilum clamping, and 26.6% required CPB. It is not surprising that 89.5% of our patients endured intraoperative hypotension (mean arterial pressure < 65 mmHg) with a median duration of 17 min (IQR 4–48) and 62.2% developed tachycardia during surgery. All these might had led to significant myocardial injury [34–36].

In our patients, the majority of myocardial injury was silent (without ischemic symptoms, ECG changes or imaging findings) and occurred within the first day after surgery. These were in line with previous studies [17, 18, 33]. We found that combined Mayo level III + IV was an independent risk factor of postoperative myocardial injury when compared with combined level I + II, possibly due to more severe intraoperative hemodynamic fluctuation. In line with this, previous studies also reported that patients with higher level tumor thrombus (usually level III or higher) had increased major complications and even mortality [15, 37, 38]. Therefore, patients with IVC thrombus of level III or higher should be managed more cautiously. In our results, long duration tachycardia was another independent risk factor of myocardial injury. This could be explained by increased oxygen demand and shortened diastolic phase of the myocardium; the underlying hemodynamic changes might also contribute. The association between tachycardia and myocardial injury had been reported in other surgical patients [35, 36]. As a potentially modifiable factor, intraoperative tachycardia should be prevented and carefully managed. We found that acute normovolemic hemodilution was an independent risk factor of myocardial injury. This may be explained by the decrease of oxygen content and delivery after hemodilution [39, 40]. Whereas 62.2% of our patients developed tachyarrhythmia which increased oxygen consumption and, thus, myocardial oxygen delivery-consumption mismatch. In line with others [41], long duration of surgery was also identified as a risk factor of myocardial injury in this cohort. On the other hand, we identified male sex as a protective factor. One possible reason was that large vessel size in male patients (compared with females) made the procedures less difficult. In accord with ours, Hassan and colleges [42] reported that female gender was an independent risk of major adverse cardiovascular events after peripheral artery disease intervention.

The incidence of AKI in our patients was much higher than in many previous studies [24, 25, 27, 28]. But our results were close to those of Shin et al. [30], the only study performed in a similar patient population, i.e., following nephrectomy with IVC thrombectomy. The high AKI incidence can be attributed to the type of surgery (radical nephrectomy) [26] and the concomitant hemodynamic fluctuation during surgery [34, 43]. Furthermore, clamping of IVC and/or hepatic hilum during surgery might directly impair renal function [44]. In our patients, high BMI was independently associated with an increased risk of AKI. Similar association was reported in critically ill patients [45–47]. The potential mechanisms remain unclear and may include chronic inflammation associated with obesity [48]; but it is also possibly due to the method defining AKI, i.e., patients with high body mass produce more creatinine. We found that long duration of intraoperative hypotension was an independent risk factor of AKI; this was in line with previous findings [34, 49, 50]. Optimizing blood pressure management may improve outcome but requires further confirmation [51]. On the contrary, we found that preoperative renal arterial embolism was a protective factor of AKI. The potential advantages of renal artery embolism in patients with IVC tumor thrombus had been reported by others [52]. Our results provide further evidence although more studies are required.

In addition to the retrospective nature, our study had some other limitations. First, because of the rarity of the surgery, the sample size included in the study was relatively small. We might have missed some risk factors. Second, we only collected in-hospital data. Long-term outcomes of our patients remained unclear. Third, as a single-center study, the generalizability of our results may be limited. Fourth, there might be interaction between myocardial injury and AKI in our patients. However, because of limited sample size and follow-up duration, we did not evaluate heart-renal cross-talk in this study [53].

In summary, our results showed that myocardial injury and AKI were common in patients recovering from radical nephrectomy with IVC thrombectomy. Patients who developed these complications had worse perioperative outcomes. Among the potentially modifiable factors, long-duration intraoperative tachyarrhythmia was associated with increased risk of myocardial injury; preoperative renal artery embolism was associated with a decreased risk, whereas long-duration intraoperative hypotension was associated with an increased risk of AKI. Further studies are required to investigate whether intervention targeting these factors can improve patients’ outcomes.

## Supplementary Information

**Additional file 1.** STROBE checklist.

**Additional file 2.** Supplement Table. Univariable analyses of perioperative factors.

## Data Availability

The datasets used and analyzed in the current study are available from the corresponding author upon reasonable request.

## Abbreviations

AKI: Acute kidney injury
KDIGO: Kidney Disease: Improving Global Outcomes
OR: Odd ratio
CI: Confidence interval
RCC: Renal cell carcinoma
IVC: Inferior vena cava
ECG: Electrocardiogram
ANH: Acute normovolemic hemodilution
CPB: Cardiopulmonary bypass
ICU: Intensive care unit
cTnI: Cardiac troponin I
PCA: Patient-controlled analgesia
AMI: Acute myocardial infarction
IQR: Interquartile range
BMI: Body mass index
HR: Hazard ratio
