# Supplementary material for: Prevalence and risk factors of myocardial and acute kidney injury following radical nephrectomy with vena cava thrombectomy: a retrospective cohort study
Source: BMC Anesthesiol. 2021 Oct 12;21:243. doi: 10.1186/s12871-021-01462-y (PMC8513361; doi:10.1186/s12871-021-01462-y)
Supplement: Supplementary file 2 — Additional file 2. Supplement Table. Univariable analyses of perioperative factors. [file 12871_2021_1462_MOESM2_ESM.docx]

**Supplement Table. Univariable analyses (logistic regression analyses)**

|  | Number | Myocardial injury (n=54) | P value | Acute kidney injury (n=61) | P value |
| --- | --- | --- | --- | --- | --- |
|  |  | Odds ratio (95% CI) |  | Odds ratio (95% CI) |  |
| **Baseline/preoperative data** |  |  |  |  |  |
| Age, year | 143 | 0.97 (0.95, 1.00) | **0.069** | 1.01 (0.98, 1.04) | 0.431 |
| Body mass index, kg/ m^2^ | 143 | 0.96 (0.88, 1.04) | 0.299 | 1.12 (1.01, 1.23) | **0.029** |
| Male sex | 105 | 0.43 (0.20, 0.92) | **0.029** | 0.67 (0.32, 1.41) | 0.287 |
| Comorbidities |  |  |  |  |  |
| Stroke | 7 | 2.29 (0.49, 10.66) | 0.290 | 3.57 (0.67, 19.07) | **0.136** |
| Hypertension | 58 | 0.97 (0.51, 2.05) | 0.973 | 1.31 (0.67, 2.56) | 0.437 |
| Chronic heart disease ^a^ | 12 | 1.73 (0.53, 5.66) | 0.365 | 1.38 (0.42, 4.51) | 0.592 |
| Diabetes Mellitus | 15 | 0.91 (0.37, 2.24) | 0.841 | 0.58 (0.23, 1.44) | 0.239 |
| Chronic obstructive pulmonary disease | 4 | -- | >0.999 | -- | >0.999 |
| Elevated transaminase ^b^ | 11 | 1.41 (0.41, 4.87) | 0.585 | 0.75 (0.21, 2.69) | 0.661 |
| Abnormal kidney function ^c^ | 11 | 0.34 (0.07, 1.65) | **0.181** | 0.48 (0.12, 1.88) | 0.292 |
| Smoking history | 47 | 1.03 (0.50, 2.12) | 0.926 | 1.29 (0.64, 2.60) | 0.483 |
| Previous surgery | 25 | 1.12 (0.46, 2.71) | 0.800 | 0.46 (0.18, 1.19) | **0.108** |
| Charlson Comorbidity Index ^d^ | 143 | 0.86 (0.33, 2.18) | 0.748 | 1.04 (0.42, 2.56) | 0.931 |
| Laboratory tests |  |  |  |  |  |
| Hemoglobin, g/L | 143 | 1.00 (0.98, 1.01) | 0.679 | 1.02 (0.99, 1.01) | 0.394 |
| Albumin, g/L | 143 | 0.96 (0.89, 1.04) | 0.315 | 0.98 (0.91, 1.06) | 0.635 |
| American Society of Anesthesiologists class |  |  |  |  |  |
| I+II | 100 | Ref |  | Ref |  |
| III+IV | 43 | 1.94 (0.94, 4.02) | **0.075** | 1.25 (0.61, 2.57) | 0.541 |
| New York Heart Association class |  |  |  |  |  |
| I | 124 | Ref |  | Ref |  |
| II+III | 19 | 0.93 (0.35, 2.60) | 0.929 | 0.76 (0.28, 2.05) | 0.583 |
| Maximum tumor diameter, cm |  |  |  |  |  |
| 0 to 5 | 12 | Ref |  | Ref |  |
| >5 to 10 | 76 | 2.10 (0.52, 8.52) | 0.301 | 1.23 (0.35, 4.39) | 0.747 |
| >10 | 55 | 1.23 (0.30, 5.19) | 0.777 | 0.55 (0.15, 2.06) | 0.377 |
| Mayo classification ^e^ |  |  |  |  |  |
| I+II | 51 | Ref |  | Ref |  |
| III+IV | 92 | 8.18 (3.18, 21.05) | **<0.001** | 1.61 (0.80, 3.26) | **0.186** |
| Interval from contrast-enhanced examination, day | 143 | 1.00 (0.98, 1.03) | 0.720 | 1.02 (0.99, 1.05) | **0.144** |
| **Intraoperative data** |  |  |  |  |  |
| Selective renal arterial embolization | 27 | 0.64 (0.26, 1.59) | 0.336 | 0.40 (0.16, 1.02) | **0.056** |
| Type of anesthesia |  |  |  |  |  |
| General | 123 | Ref |  | Ref |  |
| Combined regional-general ^f^ | 20 | 0.87 (0.32, 2.34) | 0.784 | 0.56 (0.22, 1.45) | 0.233 |
| Medication during anesthesia |  |  |  |  |  |
| Nitrous oxide | 102 | 0.46 (0.22, 0.95) | **0.037** | 0.71 (0.34, 1.46) | 0.349 |
| Sevoflurane | 79 | 0.80 (0.41, 1.58) | 0.525 | 1.65 (0.84, 3.25) | **0.145** |
| dexmedetomidine | 60 | 1.17 (0.60, 2.30) | 0.648 | 0.63 (0.32, 1.23) | **0.176** |
| ANH before surgery ^g^ | 50 | 2.21 (1.09, 4.48) | **0.028** | 1.58 (0.79, 3.17) | **0.194** |
| Extremes of arterial blood gas analysis |  |  |  |  |  |
| Lowest hemoglobin, g/L | 143 | 0.96 (0.95, 0.98) | **<0.001** | 1.00 (0.99, 1.02) | 0.629 |
| Highest lactic acid, mmol/L | 143 | 1.93 (1.29, 2.89) | **0.001** | 1.38 (0.98, 1.95) | **0.066** |
| Hemodynamic change during anesthesia |  |  |  |  |  |
| Hypotension ^h^ | 128 | 4.45 (0.96, 20.54) | **0.056** | 1.13 (0.38, 3.36) | 0.826 |
| Duration of hypotension, 20 min | 143 | 1.37 (1.11, 1.69) | **0.004** | 1.27 (1.04, 1.54) | **0.021** |
| Tachycardia ^i, j^ | 89 | 4.30 (1.93, 9.60) | **<0.001** | 1.87 (0.93, 3.77) | **0.081** |
| Duration of tachycardia, 20 min | 143 | 1.73 (1.18, 2.53) | **<0.001** | 0.99 (0.79, 1.24) | 0.990 |
| Bradycardia ^k^ | 27 | 2.99 (1.26, 7.06) | **0.013** | 1.32 (0.57, 3.05) | 0.523 |
| Fluid infused, 100 ml | 143 | 1.02 (1.01, 1.04) | **0.004** | 0.99 (0.98, 1.01) | 0.294 |
| Crystalloid, 100 ml | 143 | 1.02 (0.99, 1.04) | 0.218 | 0.98 (0.96, 1.00) | 0.980 |
| Artificial colloid, 100 ml | 143 | 1.04 (0.99, 1.09) | **0.147** | 0.98 (0.94, 1.03) | 0.459 |
| Hydroxyethyl starch, 100 ml | 143 | 1.04 (0.96, 1.11) | 0.356 | 1.02 (0.95, 1.09) | 0.674 |
| Succinylated gelatin, 100 ml | 143 | 1.03 (0.97, 1.09) | 0.329 | 0.97 (0.92, 1.03) | 0.363 |
| Urine output, 100 ml | 143 | 1.10 (1.04, 1.16) | **<0.001** | 0.97 (0.93, 1.02) | 0.276 |
| Allogeneic blood transfusion | 81 | 5.25 (2.40,11.49) | **<0.001** | 1.33 (0.68, 2.61) | 0.404 |
| Volume of red blood cell, ml | 143 | 1.00 (1.00, 1.01) | 0.687 | 1.03 (0.91, 1.17) | 0.637 |
| Fresh frozen plasma | 62 | 3.70 (1.82, 7.54) | **<0.001** | 1.20 (0.61, 2.34) | 0.596 |
| Platelet concentrate | 23 | 27.68 (6.15, 124.7) | **<0.001** | 2.42 (0.97, 6.03) | **0.059** |
| Intraoperative fluid balance, 100 ml | 143 | 1.01 (1.00, 1.03) | **0.156** | 0.99 (0.97, 1.01) | 0.340 |
| Duration of anesthesia, h | 143 | 1.74 (1.40, 2.15) | **<0.001** | 1.09 (0.86, 1.17) | 0.963 |
| Type of surgery | 143 |  |  |  |  |
| Laparoscopic | 19 | Ref |  | Ref |  |
| Open | 61 | 0.31 (0.81, 1.18) | 0.286 | 1.27 (0.65, 2.58) | 0.230 |
| Laparoscopic + open | 63 | 0.45 (0.65, 2.71) | 0.446 | 1.45 (0.74, 3.15) | 0.255 |
| Combined with non-renal surgery ^l^ | 3 | 0.82 (0.07, 9.27) | 0.821 | 0.85 (0.57, 10.0) | 0.999 |
| Complete inferior vena cava clamping | 119 | 3.62 (1.17, 11.3) | **0.026** | 1.05 (0.43, 2.55) | 0.914 |
| Hepatic hilum clamping | 39 | 9.16 (3.93, 21.4) | **<0.001** | 0.79 (0.37, 1.67) | 0.535 |
| Use of cardiopulmonary bypass | 38 | 20.12 (7.47, 54.2) | **<0.001** | 1.30 (0.62, 2.74) | 0.494 |
| Duration of cardiopulmonary bypass, min | 143 | 1.12 (1.07, 1.18) | **<0.001** | 1.01 (0.99, 1.03) | 0.408 |
| Duration of surgery, h | 143 | 1.71 (1.37, 2.12) | **<0.001** | 1.04 (0.88, 1.22) | 0.654 |
| Use of patient-controlled analgesia after surgery | 135 | --- | >0.999 | 5.60 (0.67, 46.78) | **0.112** |
| Postoperative fluid balance, ml |  |  |  |  |  |
| Postoperative day 1 | 143 | 0.99 (0.98, 1.01) | 0.351 | 1.01 (0.99, 1.03) | 0.276 |
| Postoperative day 2 | 143 | 1.01 (0.98, 1.05) | 0.396 | 1.02 (0.99,1.05) | 0.219 |
| Use of NSAIDs during perioperative period | 143 | 0.19 (0.09, 0.42) | **<0.001** | 1.03 (0.53, 2.01) | 0.926 |

ANH, acute normovolemic hemodilution. P values in bold indicate <0.20.

^a^ Including coronary artery disease, cardiac valve disease, or any type of arrhythmia requiring therapy.

^b^ Serum aspartate aminotransferase and/or alanine aminotransferase were higher than the upper normal limit.

^c^ Indicating serum creatinine ≥133 μmol/L.

^d^ According to the 1987 version without age correction.

^e^ Level I, tumor thrombus extending into the IVC to no more than 2 cm above the renal vein; level II, thrombus extending into the IVC to more than 2 cm above renal vein but below the hepatic veins; level III, thrombus extending into the IVC to above the hepatic vein but not to the diaphragm; and level IV, thrombus extending into the supradiaphragmatic IVC or right atrium

^f^ Includes epidural anesthesia or rectus sheath/transversus abdominis plane block.

^g^ Performed after anesthesia induction through central venous line.

^h^ Mean arterial pressure <65 mmHg.

^i^ Heart rate >100 beats per minute.

^j^ New onset arrhythmia requiring therapy, including atrial premature, ventricular premature, atrial fibrillation, etc. These patients developed tachycardia simultaneously.

^k^ Heart rate <50 beats per minute.

^l^ Combined with splenectomy or cholecystectomy.
